# Identification of Minor Benzoylated 4-Phenylcoumarins from a *Mammea neurophylla* Bark Extract

**DOI:** 10.3390/molecules201017735

**Published:** 2015-09-25

**Authors:** Bach Tai Dang, Caroline Rouger, Marc Litaudon, Pascal Richomme, Denis Séraphin, Séverine Derbré

**Affiliations:** 1 EA 921 SONAS, Université D’Angers, SFR QUASAV 4207, Campus du Végétal, 42, rue Georges Morel, Beaucouzé 49070, France; E-Mails: taihoa1@gmail.com (B.T.D.); caroline.rouger@univ-angers.fr (C.R.); pascal.richomme@univ-angers.fr (P.R.); denis.seraphin@univ-angers.fr (D.S.); 2 Institut de Chimie des Substances Naturelles, CNRS UPR 2301, Université Paris-Saclay, 1, av. de la Terrasse, Gif-sur-Yvette 91198, France; E-Mail: marc.litaudon@cnrs.fr

**Keywords:** 4-(1-acetoxypropyl)coumarins, benzoylcoumarins, *Calophyllaceae*, dereplication analysis, 4-phenylcoumarins

## Abstract

Through dereplication analysis, seven known Mammea coumarins were identified in a fraction obtained from *Mammea neurophylla* dichloromethane bark extract selected for its ability to prevent advanced glycation end-product (AGE) formation. Among them, a careful examination of the NMR dataset of pedilanthocoumarin B led to a structural revision. Inspection of LC-DAD-MS^n^ chromatograms allowed us to predict the presence of four new compounds, which were further isolated. Using spectroscopic methods (^1^H-, ^13^C- and 2D-NMR, HRMS, UV), these compounds were identified as new benzoyl substituted 4-phenylcoumarins (iso-pedilanthocoumarin B and neurophyllol C) and 4-(1-acetoxypropyl)coumarins cyclo F (ochrocarpins H and I).

## 1. Introduction

The vascular endothelium is the innermost layer of cells in vessels. In diabetes, chronic hyperglycemia induces advanced glycation end-product (AGE) formation. Their accumulation in tissues and blood contributes to endothelial dysfunction [1,2] and, consequently, to disorders, such as atherosclerosis, cardiovascular events and graft rejection, through the expression of inflammatory and immune mediators on the endothelial cell surface [3].

An anti-AGE screening of different plant extracts obtained from *Clusiaceae* and *Calophyllaceae* species previously led to selecting *Mammea neurophylla* (Schltr.) Kosterm, an endemic neocaledonian shrub, for further phytochemical and biological investigations [4,5]. In the dichloromethane (DCM) bark extract, Mammea coumarins were demonstrated to be responsible for the anti-AGE, anti-inflammatory and vasorelaxing effects [5]. This paper deals with the study of minor compounds that were not investigated during the aforementioned preliminary study.

## 2. Results and Discussion

The *M. neurophylla* DCM bark extract was fractionated using flash chromatography to give 26 fractions, which were tested for their anti-AGE properties [5,6]. Among them, Fraction VIII exhibited even a stronger anti-AGE potential than the natural reference compound quercetin (IC_50_ 0.04 and 0.06 mg/mL, respectively). Therefore, this fraction was first analyzed following a rapid, sensitive and simple dereplication method of Mammea coumarins using LC-DAD-MS^n^ that we recently published (Figure S1) [7].

### 2.1. Dereplication of Known Mammea Coumarins

By comparison of UV spectra, as well as mass spectra and fragmentations (Table 1) and by interlocking literature data via the Scifinder database [8], seven known coumarins were presumably detected in the fraction and associated with peaks 8.1 to 8.11 (Figure S1). As far as peaks 8.1, 8.4 and 8.8 to 8.11 were concerned, mass fragmentation of the quasi-molecular peak in positive ionization mode suggested a 4-(1-acetoxypropyl)coumarin (Mammea E type coumarins), as a loss of both 42 and 60 u was observed to form a stable ion. In negative mode, the loss of 60 u confirmed this substitution. Their UV spectrum (λ_max_ = 222, 295, sh 325–330 nm) indicated 4-alkyl-5,7-dihydroxycoumarins exhibiting an 8-acyl substituent [9,10,11]. For 8.10 and 8.11, mass fragmentations were typical of prenylated Mammea E with fragments at *m*/*z* 315 [M + H − 60 − 56]^+^. Thus, considering their hypothetical molecular mass (430 g·mol^−1^) and retention time, a 3-methyl-1-oxobutyl or a 2-methyl-1-oxobutyl could be proposed at C-8 to suggest Mammea E/BA (**1**) and Mammea E/BB (**2**) for peaks 8.11 and 8.10, respectively [7]. For peaks 8.8 and 8.9, losses of 42 and 60 u in positive ionization mode were preceded by a water loss, suggesting a substitution by a 2-hydroxy-3-methylbut-3-enyl group at C-6. Considering their molecular mass (446 g·mol^−1^), as well as the loss of 86 u of the base peak [M + H − H_2_O]^+^ in positive mode, the acyl group at C-8 should be a 3-methyl-1-oxobutyl or a 2-methyl-1-oxobutyl. Peaks 8.9 and 8.8 were therefore hypothesized to be neurophyllol A (**3**) and neurophyllol B (**4**). The structures of these four coumarins were confirmed by NMR experiments after purification steps [4,12]. Finally, as fragmentations for peaks 8.1 and 8.4 were identical with those observed for Mammea cyclo F coumarins (Figure S2) [7], they were identified as ochrocarpin F (**5**) and G (**6**), which was confirmed by NMR spectral analysis after isolation [13].

The UV spectrum of peak 8.6 (λ_max_ = 255, 313 nm) showed a bathochromic shift in comparison to 4-phenylcoumarins, usually substituted by an acyl group at C-6 or C-8, suggesting a less common substitution at this position. Such a UV spectrum could correspond to a 4-phenylcoumarin substituted with a benzoyl group at position 8 [14]. For these Mammea A coumarins, the quasi-molecular ion ([M + H]^+^ or [M − H]^−^) usually appeared as the base peak in both ionization modes. Thus, 426 g·mol^−1^ was proposed as a hypothetical molecular mass. The loss of 56 u to form a stable ion in positive ionization mode corresponds to a non-cyclized 4-phenylcoumarins bearing a prenyl group. The revised structure of pedilanthocoumarin B (**7**) [14] (see below) was attributed to peak 8.7 (λ_max_ = 255, 301 nm), whereas peak 8.6 was considered as associated with a new compound.

### 2.2. Structure Prediction of Structures of Original Mammea Coumarins

**Figure 1:**
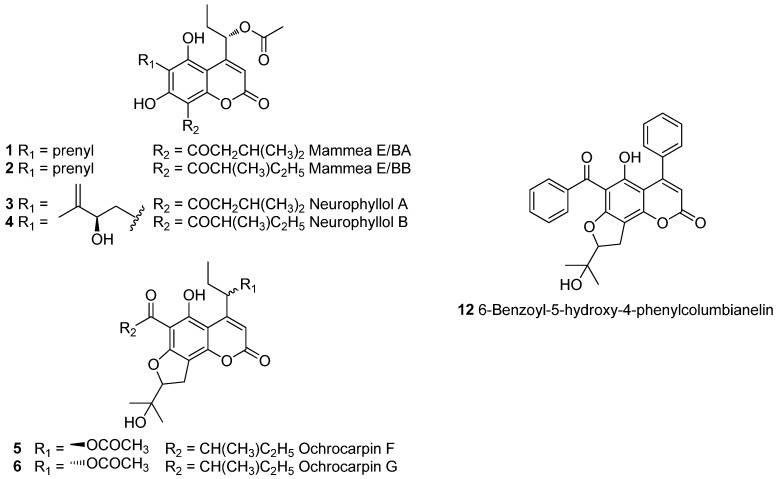
Structure of known coumarins **1** to **6** and **12** isolated from *Mammea neurophylla* bark.

**Figure 2:**
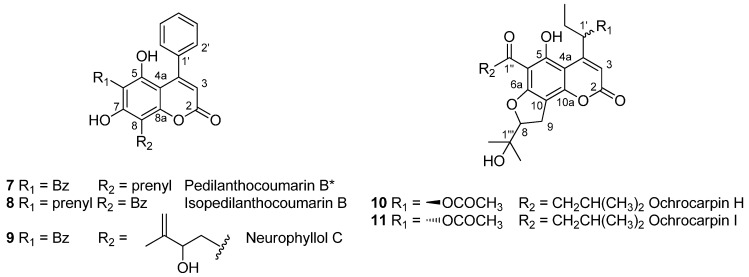
Structure of new or revised coumarins **7** to **11** isolated from *Mammea neurophylla* bark. * Revised structure of pedilanthocoumarin B.

In addition to seven known coumarins (Figure 1), five previously unreported or revised compounds (**7** to **11**) were detected (Figure 2).

**Table 1:** Retention time, (*t*_R_), UV spectrum, HRMS and ESI-MS^n^ data for peaks identified in Fraction VIII from dichloromethane (DCM) bark extract.

Peak	*t*_R_ (min)	UV λ_max_ (nm)	(+)-ESI-MS *m/z*	(+)-ESI-MS^2^ *m/z*	(−)-ESI-MS *m/z*	(−)-ESI-MS^2^ *m/z*	Hypothetical Molecular Mass (g·mol^−1^)	Hypothetical Structure ^1^
8.1	11.0	222, 299	447 [M + H]^+^ 469 [M + Na]^+^	429/405/387/375/369/361/351/343/315	445 [M − H]^−^	385/327	446	Ochrocarpin F (**5**)
8.2	12.4	222, 299	447 [M + H]^+^	429/405/387/369/361/351/343/315	445 [M − H]^−^	385/327	446	New product: ochrocarpin H (**10**)
8.3	13.0	255, 298	425 [M − H_2_O + H]^+^	407/347	441 [M − H]^−^	423/369	442	New product: neurophyllol C (**9**) 6-benzoyl-5-hydroxy-4-phenylcolumbianelin (**12**)
8.4	14.3	222, 299	447 [M + H]^+^ 469 [M + Na]^+^	429/405/387/375/369/361/351/343/315	445 [M − H]^−^	385/327	446	Ochrocarpin G (**6**)
8.5	15.9	222, 298	447 [M + H]^+^	429/405/387/375/369/361/351/343/315	445 [M − H]^−^	385/327	446	New product: ochrocarpin I (**11**)
8.6	18.9	255, 313	427 [M + H]^+^	371	425 [M − H]^−^	347	426	New product: Iso-pedilanthocoumarin B (**8**) *
8.7	20.1	256, 301	427 [M + H]^+^	371	425 [M − H]^−^	347	426	Pedilanthocoumarin B (**7**)
8.8	26.6	222, 295, 330	429 [M + H − H_2_O]^+^ 447 [M + H]^+^ 469 [M + Na]^+^	387/369/351/343	445 [M − H]^−^	385	446	Neurophyllol B (**4**)
8.9	28.2	222, 295, 330	429 [M + H − H_2_O]^+^ 447 [M + H]^+^ 469 [M + Na]^+^	387/369/343	445 [M − H]^−^	385	446	Neurophyllol A (**3**)
8.10	33.9	222, 295, 335	431 [M + H]^+^ 453 [M + Na]^+^	389/371/315	429 [M − H]^−^	369	430	Mammea E/BB (**2**)
8.11	35.7	222, 295, 335	431 [M + H]^+^ 453 [M + Na]^+^	389/371/315	429 [M − H]^−^	369	430	Mammea E/BA (**1**)

^1^ These structures were confirmed after purification and NMR experiments; * structure previously known as pedilanthocoumarin B.

Compound **8** (peak 8.6) exhibited similar mass spectra and fragmentation patterns as pedilanthocoumarin B (**7**), suggesting a 4-phenylcoumarin with the same substituents, *i.e.*, one prenyl and one benzoyl moiety. As a bathochromic shift was observed on the UV spectrum (λ_max_ = 255, 313 nm), **8** was hypothesized as iso-pedilanthocoumarin B, a 4-phenylcoumarin bearing a prenyl substituent at C-6 and a benzoyl group at C-8. Compound **9** (peak 8.3) showed a similar UV spectrum as pedilanthocoumarin B **7**, suggesting a 4-phenylcoumarins with a benzoyl group at C-6. As observed for neurophyllols **3** to **4**, the base peak in positive ionization mode [M + H − H_2_O]^+^ corresponded to a water loss and was followed by either an additional water loss (−18 u) or the fragmentation of the pyran ring (−60 u). This suggested that the Mammea A coumarin was substituted by a 2-hydroxy-3-methylbut-3-enyl moiety at C-8 [7]. To our knowledge, neither **8** nor **9** have been previously identified.

The structures of Compounds **10** and **11** (peaks 8.2 and 8.5) seemed very close to those of ochrocarpin F (**5**) and G (**6**). Indeed, no differences were noticed on UV spectra, as well as mass spectra and fragmentations. As it was previously described that Mammea cyclo E and cyclo F show similar fragmentation pathways [7], **10** and **11** were hypothesized as 4-(1-acetoxypropyl)coumarins cyclo E or cyclo F substituted by a 3-methyl-1-oxobutyl or a 2-methyl-1-oxobutyl.

### 2.3. Purification and Structural Analysis of Coumarins **7** to **12**

To verify our hypothesis, Fraction VIII was fractionated using flash chromatography, and Compounds **7** to **12** were purified by preparative HPLC.

#### 2.3.1. Structure Revision of Pedilanthocoumarin B (**7**)

^1^H- and ^13^C-NMR data of **7** were identical to those previously reported by Sandjo *et al.*, for pedilanthocoumarin B [14]. Indeed, pedilanthocoumarin B was identified as a 4-phenylcoumarin substituted by a prenyl group at C-6 and a benzoyl group at C-8 based on HMBC correlations between the prenyl methylene at δ_H_ 3.56 and the carbons at δ_c_ 109.2, 157.4 and 160.9, respectively assigned by the authors to C-6, C-5 and C-7. However, since C-8a also has a chemical shift at 157.4, carbons at δ_c_ 109.2, 157.4 and 160.9 could also be assigned to C-8, C-8a and C-7 from the same correlations. In our study, the ^1^H-NMR spectrum of Compound **7**, recorded in CDCl_3_ between 0 and 20 ppm, showed two hydroxyl signals at δ_H_ 8.60 and 9.05 ppm. They were respectively attributed to 5-OH and 7-OH, as indicated by a correlation between δ_H_ 9.05 and δ_c_ 109.2 on the HMBC spectrum. No signal corresponding to a strongly-chelated hydroxyl was observed, as expected for a 7-OH/8-benzoyl pattern [12,15]. These key features were not discussed in the first publication on pedilanthocoumarin B (**7**) [14]. Moreover, as explained below, NMR data for Compound **8** unambiguously led to a 6-prenyl-8-benzoylcoumarin. Consequently, Compound **7** was identified as pedilanthocoumarin B, but with a structure now revised as a 4-phenylcoumarin substituted by a benzoyl group at C-6 and a prenyl group at C-8.

#### 2.3.2. Iso-Pedilanthocoumarin B (**8**)

**Figure 3:**
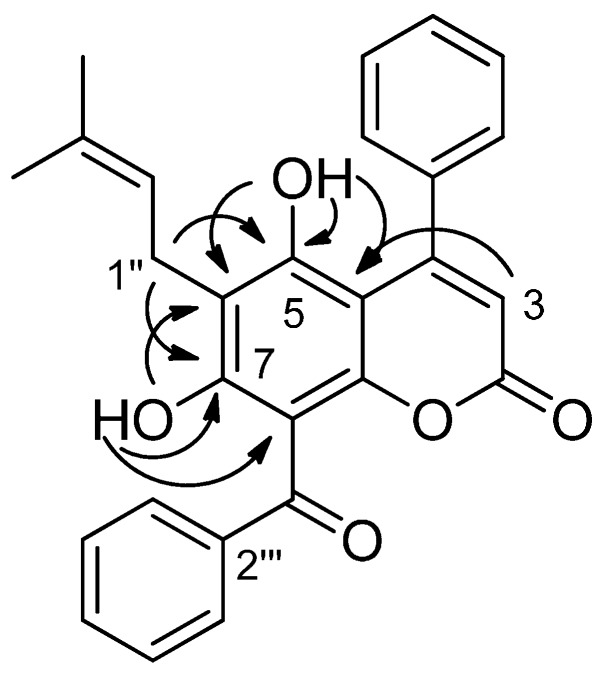
Selected HMBC correlations for iso-pedilanthocoumarin B (**8**).

The HRESIMS of Compound **8** exhibited a pseudomolecular ion [M + H]^+^ at *m*/*z* 427.1545, which corresponds to the molecular formula C_27_H_23_O_5_ (calcd. for [M + H]^+^: 427.1540). LC-DAD-MS^n^ analyses suggested a 4-phenylcoumarin bearing a prenyl substituent at C-6 and a benzoyl group at C-8, as explained previously in Section 2.2. This was confirmed by the NMR spectra of the pure product. Similarly to pedilanthocoumarin B (**7**), the ^1^H-NMR spectrum (Table 2) exhibited signals for five aromatic protons (δ_H_ 7.44 (H-2′, H-4′ and H-6′) and δ_H_ 7.57 (H-3′ and H-5′)) and the typical singlet (δ_H_ 5.89) for the hydrogen of an α-pyrone ring system (H-3). These elements together with the long-range coupling observed in the HMBC spectrum between H-3 and the quaternary aromatic carbon (δ_C_ 136.5 (C-1′)) pointed to a 4-phenylcoumarin structure. Five additional aromatic protons (δ_H_ 7.48 (H-4‴ and H-6‴), 7.59 (H-5‴) and δ_H_ 7.66 (H-3‴ and H-5‴)) were attributed to a benzoyl group, as an HMBC correlation between H-3‴/H-5‴ (7.66 ppm) and C-1‴ (199.1 ppm) was observed. The presence of a prenyl group was detected by the signals at δ_H_ 3.35 (H-1″), δ_H_ 5.16 (H-2″), δ_H_ 1.73 (Me-4″) and δ_H_ 1.68 (Me-5″). Thus, NMR data confirmed this compound to be an isomer of pedilanthocoumarin B (**7**). The presence of two hydroxyls, a chelated one at δ_H_ 12.34 and another one at δ_H_ 6.00, was also noticed. Their chemical shifts, different from pedilanthocoumarin B (**7**), and HMBC experiments (Figure 3 and Figure S7) ascertained the position of the prenyl group at C-6 and the benzoylation at C-8. Particularly, correlations between 5-OH (6.00 ppm)/H-3 (5.89 ppm) and C-4a (100.8 ppm) or between H-1″ (3.35 ppm) and C-5 (157.3 ppm)/C-7 (164.4 ppm) were observed. Therefore, **8** is a new coumarin named iso-pedilanthocoumarin B.

**Table 2:** ^1^H- (500 MHz, CDCl_3_) and ^13^C-NMR (125 MHz, CDCl_3_) data for pedilanthocoumarin B (**7**), iso-pedilanthocoumarin B (**8**) and neurophyllols C (**9**).

Position	Pedilanthocoumarin B (7)		Neurophyllol C (9)		Position	Iso-pedilanthocoumarin B (8)	
	δ_C_	δ_H_ (*J* in Hz)	δ_C_	δ_H_ (*J* in Hz)		δ_C_	δ_H_ (*J* in Hz)
2	159.7		159.9		2	158.1 ^b^	
3	113.3	6.01, s	112.7	6.01, s	3	113.0	5.89, s
4	154.6		156.3		4	152.2 ^b^	
4a	101.6		102.3		4a	100.8 ^b^	
5	157.4		160.5		5	157.3 ^b^	
6	107.3		108.2		6	112.3 ^b^	
7	160.9		161.1		7	164.4 ^b^	
8	109.2		105.6		8	104.3 ^b^	
8a	157.4		157.8		8a	nd	
1′	137.6		139.1 ^a^		1′	136.5 ^b^	
2′, 6′	127.5	7.37, m	127.4	7.36, m	2′, 6′	127.7	7.44, m
3′, 5′	129.0 ^a^	7.44, m	128.0 ^a^	7.43, m	3′, 5′	129.8	7.57, m
4′	129.7	7.44, m	128.6	7.43, m	4′	130.4 ^b^	7.44, m
1″	199.1		200.0		1″	21.9	3.35, d (7.0)
2″	139.8		140.7 ^a^		2″	120.9	5.16, t (7.0)
3″, 7″	128.6	7.63, m	128.6	7.64, m	3″	134.2 ^b^	
4″, 6″	128.6 ^a^	7.44, m	128.1 ^a^	7.41, m	4″	18.0	1.73, s
5″	132.9	7.56, m	132.2	7.52, m	5″	25.9	1.68, s
1‴	22.0	3.56, d (7.0)	28.7	3.07, dd, (15.0, 8.0) 3.26, dd (15.0, 2.5)	1‴	199.1 ^b^	
2‴	120.8	5.27, td (7.0, 1.5)	76.7	4.48, dd (8.0, 2.5)	2‴	140.5 ^b^	
3‴	134.8		146.2		3‴, 7‴	128.4	7.66, m
4‴	18.2	1.84, s	111.1	4.91, s 4.97, s	4‴, 6‴	127.7	7.48, m
5‴	26.0	1.71, s	18.9	1.90, s	5‴	132.5	7.59, m
5-OH		8.60, s		11.69, s	5-OH		6.00, s
7-OH		9.05, s		9.57, s	7-OH		12.34, s

^a^ Interchangeable; ^b^ chemical shifts deduced from the HMBC spectrum; nd not determined.

#### 2.3.3. Neurophyllol C (**9**)

The HRMALDI-TOFMS of Compound **9** exhibited a pseudomolecular ion [M − H]^−^ at *m*/*z* 441.1355, which corresponds to the molecular formula C_27_H_23_O_6_ (calcd. for [M − H]^−^: 441.1344). LC-DAD-MS^n^ analyses suggested a 4-phenylcoumarin bearing a benzoyl group at C-6 and a 2-hydroxy-3-methylbut-3-enyl moiety at C-8. This was confirmed by the NMR dataset of **9**, which appeared similar to pedilanthocoumarin B (**7**), except that no signal corresponding to a prenyl group was observed. Instead, the presence of a 2-hydroxy-3-methylbut-3-enyl moiety at C-8 was deduced from characteristic signals [15]: two methylenic proton signals at δ_H_ = 3.07 (1H, dd, *J* = 8.0 and 15.0 Hz, H-1‴) and 3.26 (1H, dd, *J* = 2.5 and 15.0 Hz, H-1‴), an oxymethine proton signal at δ_H_ = 4.48 (1H, dd, *J* = 2.5 and 8.0 Hz, H-2‴), two olefinic *gem*-proton signals at δ_H_ = 4.91 and 4.97 (each 1H, s, H-4‴) and, finally, a methyl proton signal at δ_H_ = 1.90 (3H, s, H-5‴). The presence of this substituent was confirmed by ^13^C-NMR with specific δ_C_ at 28.7 (C-1‴), 76.7 (C-2‴), 146.2 (C-3‴), 111.1 (C-4‴) and 18.9 (C-5‴) (Table 2). The signals of two hydroxyls at δ_H_ 11.69 and 9.57 ppm were noticed. They were respectively attributed to 5-OH and 7-OH, as indicated by correlations between 5-OH (11.69 ppm)/H-3 (6.01 ppm) and C-4a (102.3 ppm) on the HMBC spectrum. HMBC experiments ascertained the position of the 2-hydroxy-3-methylbut-3-enyl moiety at C-8 and, consequently, the benzoyl group at C-6. Particularly, correlations between H-1‴ (3.07 and 3.26 ppm) and C-7 (161.1 ppm)/C-8 (105.6 ppm)/C-8a (157.8 ppm) were observed. Therefore, **9** was named neurophyllol C.

#### 2.3.4. Ochrocarpin H (**10**) and Ochrocarpin I (**11**)

^1^H- and ^13^C-NMR data for **10** and **11** appeared to be very close to those described for ochrocarpin F (**5**) and G (**6**), respectively [13]. Indeed **10** and **11** exhibited a 3-methylbutyryl group instead of a 2-methylbutyryl group at C-6, as described in Table 3. Compounds **10** and **11** were thus named ochrocarpins H and I.

**Table 3:** ^1^H- (500 MHz, CDCl_3_) and ^13^C-NMR (125 MHz, CDCl_3_) data for ochrocarpin H (**10**) and ochrocarpin I (**11**).

Position	Ochrocarpin H (10)		Ochrocarpin I (11)	
	δ_C_, Type	δ_H_ (*J* in Hz)	δ_C_, Type	δ_H_ (*J* in Hz)
2	159.5		159.6	
3	106.2	6.22, s	106.6	6.24, s
4	156.1		156.3	
4a	97.3		97.4	
5	163.5		163.6	
6	105.1		105.4	
6a	161.5		161.6	
8	93.3	4.93, t (9.0)	93.3	4.91, t (9.0)
9	26.9	3.17, m	26.7	3.19, d (9.0)
10	110.3		110.5	
10a	157.6		157.5	
1′	72.7	6.30, dd (8.0, 3.5)	72.6	6.48, dd (8.5, 3.5)
2′	28.3	1.74, m	28.8	1.75, m
3′	9.9	1.04, m	10.3	1.01, t (7.5)
OCO*CH_3_*	21.1	2.17, s	21.1	2.15, s
O*CO*CH_3_	170.3		170.8	
1″	205.9		206.2	
2″	53.6	3.12, d (6.5)	53.6	3.12, d (6.5)
3″	25.7	2.26, m	25.7	2.26, m
4″	22.8	1.03, d (6.5)	22.8	1.04, m
5″	22.8	1.03, d (6.5)	22.8	1.04, m
1‴	71.5		71.8	
2‴	25.0	1.29, s	24.3	1.26, s
3‴	26.2	1.38, s	26.4	1.39, s
5-OH		14.23, s		14.25, s

#### 2.3.5. 6-Benzoyl-5-hydroxy-4-phenylcolumbianelin (**12**)

Using UV (λ_max_ = 299 nm), HRMS and NMR data, Compound **12** was identified as 6-benzoyl-5-hydroxy-4-phenylcolumbianelin [16,17]. As for pedilanthocoumarin B (**7**) and neurophyllol C (**9**), this 4-phenylcoumarin bears a benzoyl group at C-6. Both neurophyllol C (**9**) and 6-benzoyl-5-hydroxy-4-phenylcolumbianelin (**12**) could biosynthetically derive from an oxidized pedilanthocoumarin B (**7**).

## 3. Experimental Section

### 3.1. Plant Material

As previously described, *Mammea neurophylla* bark was collected in November 1998 in the dry forest of “Conservatoire botanique de la forêt de Tiéa”, North Province (New Caledonia). A voucher specimen (LIT-0660) was deposited at the Herbarium of the Botanical and Tropical Ecology, Department of the Institut de Recherche pour le Développement (IRD) Center, Noumea (New Caledonia) [4].

### 3.2. Chemistry

#### 3.2.1. Extraction and Fractionation

As described by Dang *et al.* [5], air-dried and powdered bark (695 g) was extracted with DCM (2.5 L, 72 h) in a Soxhlet apparatus. The DCM crude extract was concentrated under vacuum at 40 °C to yield 29 g of dry extract. DCM bark extract (18 g) was subjected to flash chromatography using a PuriFlash PF-50SiHC/300G cartridge (Interchim, Clichy, France) eluted with C_6_H_12_/EtOAc (95:5 *v*/*v* to 70:30 *v*/*v* for 120 min, 50 mL/min) to afford 26 fractions, namely FI to FXXVI, in elution order.

#### 3.2.2. Fraction VIII Profiling

LC-PDA-ESIMS analyses were performed on a Waters 2795 apparatus (Waters, Guyancourt, France) equipped with a Waters 2487 UV detector and coupled with an Esquire 3000 PLUS ESI ion trap mass spectrometer equipped with an electrospray source (Bruker, Wissembourg, France) assisted by the HyStar^®^ software (Bruker Daltonics, Wissembourg, France). A 10-µL sample was directly injected onto a Lichrospher 100 RP18 column (150 × 4.6 mm, 5 µm, Merck, Darmstadt, Germany). The column was set at 20 °C. Separation was achieved using an acidic water/MeOH system. The mobile phase was as follows: water (HCOOH-0.1%)/MeOH from 30:70 to 20:80 *v*/*v* in 45 min. The flow rate was 1 mL/min with a split of 90% before mass analysis. For the study, the wavelength was set at λ = 290 nm. For each peak of interest, the UV spectrum was obtained by scanning the sample in the range of 210 to 400 nm. The mass analyses were performed in both positive and negative modes using the conditions described by Dang *et al.* [7].

#### 3.2.3. Isolation of Mammea Coumarins from Fraction VIII

Fraction VIII (170 mg) was subjected to reverse flash chromatography using a PuriFlash PF-50C18HP/35G cartridge (Interchim) eluted with 0.1% HCOOH in water/MeOH (30:70 *v*/*v* to 20:80 *v*/*v* for 135 min, 20 mL/min) to obtain pedilanthocoumarin B (**7**) (7 mg) and a mixture (43/57) of Mammea E/BA (**1**) and Mammea E/BB (**2**) (28 mg) [12]. The residue (130 mg) was purified using a GraceResolv™ Silica/24G cartridge (Grace Davison Discovery Sciences, Epernon, France) eluted with C_6_H_12_/EtOAc (100:0 *v*/*v* to 75:25 *v*/*v* for 85 min, 16 mL/min) to afford 7 fractions (VIII-1 to VIII-7). Fraction VIII-4 was a mixture (17/83) of neurophyllol A (**3**) and neurophyllol B (**4**) (12 mg) [5], and Fraction VIII-6 contained pure neurophyllol C (**9**) (6 mg).

Fraction VIII-3 (28 mg) was fractionated with RP-HPLC on a Lichrospher^®^ 100 using a mobile phase consisting of a mixture of MeOH − H_2_O + 0.1% formic acid (from 30:70 to 20:80 *v*/*v* in 45 min), flow rate at 1 mL/min, to afford pedilanthocoumarin B (**7**) (5 mg) and iso-pedilanthocoumarin B (**8**) (0.3 mg).

Fraction VIII-7 (18 mg) was subjected to a preparative HPLC on a Pursuit XRs 5 C18 column (250 × 21.2 mm, 5 µm, Agilent Technologies, Les Ulis, France), using an isocratic mobile phase consisting of a MeOH − H_2_O + 0.1% formic acid (25:75), flow rate at 15 mL/min, to afford ochrocarpins F (**5**) (4.6 mg), H (**10**) (2.2 mg), G (**6**) (3.9 mg) and I (**11**) (2.1 mg) and 6-benzoyl-5-hydroxy-4-phenylcolumbianelin (**12**) (1.1 mg) [13,17].

#### 3.2.4. Structural Analyses

^1^H-, ^13^C- and 2D-NMR spectra were recorded in CDCl_3_ on a Bruker Avance DRX 500 MHz (Bruker France, Wissembourg, France) spectrometer. Mass spectra were recorded on an ESI Esquire 3000 PLUS apparatus (Bruker), and HRMS spectra were recorded on an ESI MicrOTOF-Q II Electrospray Ionization Mass Spectrometer (Bruker) or an ESI LTQ-Orbitrap apparatus (Thermo Fisher Scientific, Les Ulis, France). Optical rotations were measured at the wavelength of the sodium D line length on a polarimeter Haensch Schmidt Polarotronic. IR spectra were recorded on a Vector 22 Bruker spectrometer, and values are reported in cm^−1^ units.

*Pedilanthocoumarin B* (**7**). Yellow solid; IR (film) ν_max_ 3440, 2930, 1710, 1598, 1450, 1196 cm^−1^; ^1^H-NMR (CDCl_3_, 500 MHz) and ^13^C-NMR (CDCl_3_, 125 MHz), see Table 2.

*Iso-pedilanthocoumarin B* (**8**). Yellow solid; ^1^H-NMR (CDCl_3_, 500 MHz) and ^13^C-NMR (CDCl_3_, 125 MHz), see Table 2; HRESIMS *m*/*z* 427.1545 [M + H]^+^ (calcd. for C_27_H_23_O_5_, 427.1540).

*Neurophyllol C* (**9**). Yellow solid; IR (film) ν_max_ 3450, 2950, 1710, 1600, 1450, 1200 cm^−1^; ${\left[\text{α}\right]}_{\text{D}}^{25}$ −0.2 [*c* 0.1, CHCl_3_]; ^1^H-NMR (CDCl_3_, 500 MHz) and ^13^C-NMR (CDCl_3_, 125 MHz), see Table 2; HRMALDI-TOFMS *m*/*z* 441.1355 [M − H]^−^ (calcd. for C_27_H_23_O_6_, 441.1344).

*Ochrocarpin H* (**10**). Red solid; IR (film) ν_max_ 3350, 1740, 1715, 1610, 1215, 1150, 1030, 955, 934 cm^−1^; ${\left[\text{α}\right]}_{\text{D}}^{25}$ −0.5 [*c* 0.04, CHCl_3_]; ^1^H-NMR (CDCl_3_, 500 MHz) and ^13^C-NMR (CDCl_3_, 125 MHz), see Table 3; HRESIMS *m*/*z* 447.2015 [M + H]^+^ (calcd. for C_24_H_31_O_8_, 447.2013).

*Ochrocarpin I* (**11**). Red solid; IR (film) ν_max_ 3405, 1735, 1710, 1610, 1215, 1145, 1035, 955, 930 cm^−1^ ${\left[\text{α}\right]}_{\text{D}}^{25}$ +0.5 [*c* 0.04, CHCl_3_]; ^1^H-NMR (CDCl_3_, 500 MHz) and ^13^C-NMR (CDCl_3_, 125 MHz), see Table 3; HRESIMS *m*/*z* 447.2014 [M + H]^+^ (calcd. for C_24_H_31_O_8_, 447.2013).

## 4. Conclusions

In conclusion, this study described the dereplicative analysis of an anti-AGE fraction from *M. neurophylla* DCM bark extract, using LC-DAD-MS^n^ data. This approach led to identifying seven well-known Mammea coumarins and to hypothesizing a new coumarinic structure for four minor compounds, as well as for pedilanthocoumarin B (**7**). After their isolation, the structures of the new products were ascertained by extensive NMR spectroscopy. Among them, three benzoylated 4-phenylcoumarins, including two new compounds, were identified for the first time in the *Mammea* genus, namely pedilanthocoumarin B (**7**), iso-pedilanthocoumarin B (**8**) and neurophyllol C (**9**). Indeed, to our knowledge, such compounds were previously isolated only in two species: *Calophyllum teysmannii* (*Calophyllaceae*) and *Pedilanthus tithymaloides* (*Euphorbiaceae*). Moreover, isolation of pedilanthocoumarin B and careful examination of NMR data led to its structural revision. Besides, ochrocarpins H and I were determined as new 4-(1-acetoxypropyl)coumarins cyclo F.

## Supplementary Materials

Supplementary materials can be accessed at: http://www.mdpi.com/1420-3049/20/10/17735/s1.

## References

[1] Brownlee M. (2001). Biochemistry and molecular cell biology of diabetic complications. Nature.

[2] Versari D., Daghini E., Virdis A., Ghiadoni L., Taddei S. (2009). Endothelium-dependent contractions and endothelial dysfunction in human hypertension. Br. J. Pharmacol..

[3] Charreau B. (2012). Signaling of endothelial cytoprotection in transplantation. Hum. Immunol..

[4] Ferchichi L., Derbré S., Mahmood K., Touré K., Guilet D., Litaudon M., Awang K., Hadi A.H.A., Le Ray A.M., Richomme P. (2012). Bioguided fractionation and isolation of natural inhibitors of advanced glycation end-products (AGEs) from *Calophyllum flavoramulum*. Phytochemistry.

[5] Dang B.T., Gény C., Blanchard P., Rouger C., Tonnerre P., Charreau B., Rakolomalala G., Randriamboavonjy J.I., Loirand G., Pacaud P. (2014). Advanced glycation inhibition and protection against endothelial dysfunction induced by coumarins and procyanidins from *Mammea neurophylla*. Fitoterapia.

[6] Séro L., Sanguinet L., Blanchard P., Dang B., Morel S., Richomme P., Séraphin D., Derbré S. (2013). Tuning a 96-well microtiter plate fluorescence-based assay to identify AGE inhibitors in crude plant extracts. Molecules.

[7] Dang B.T., Guitton Y., Freuze I., Grovel O., Litaudon M., Richomme P., Séraphin D., Derbré S. (2015). Dereplication of *Mammea neurophylla* metabolites to isolate original 4-phenylcoumarins. Phytochem. Lett..

[8] American Chemical Society—Scifinder. http//www.cas.org/products/scifinder.

[9] Crombie L., Games D.E., Haskins N.J., Reed G.F. (1972). Extractives of *Mammea americana* L. Part V: The insecticidal compounds. J. Chem. Soc. Perkin Trans..

[10] Morel C., Dartiguelongue C., Youhana T., Oger J.M., Seraphin D., Duval O., Richomme P., Bruneton J. (1999). New coumarins from *Mesua racemosa*: Isolation and synthesis. Heterocycles.

[11] Guilet D., Morel C., Noyer N., Cornec M., Seraphin D., Wiart C., Hamid A., Hadi A., Sevenet T., Richomme P. (1999). Four new 4-phenylcoumarins from *Calophyllum dispar* isolation and hemisynthesis. Heterocycles.

[12] Yang H., Protiva P., Gil R.R., Jiang B., Baggett S., Basile M.J., Reynertson K.A., Weinstein I.B., Kennelly E.J. (2005). Antioxidant and cytotoxic isoprenylated coumarins from *Mammea americana*. Planta Med..

[13] Chaturvedula V.S.P., Schilling J.K., Kingston D.G.I. (2002). New cytotoxic coumarins and prenylated benzophenone derivatives from the bark of *Ochrocarpos punctatus* from the Madagascar rainforest. J. Nat. Prod..

[14] Sandjo L.P., Foster A.J., Rheinheimer J., Anke H., Opatz T., Thines E. (2012). Coumarin derivatives from *Pedilanthus tithymaloides* as inhibitors of conidial germination in *Magnaporthe oryzae*. Tetrahedron Lett..

[15] Guilet D., Séraphin D., Rondeau D., Richomme P., Bruneton J. (2001). Cytotoxic coumarins from Calophyllum dispar. Phytochemistry.

[16] Cao S.-G., Wu X.-H., Sim K.-Y., Tan B.H.K., Vittal J.J., Pereira J.T., Goh S.-H. (1998). Minor coumarins from *Calophyllum teysmannii* var. *inophylloide* and synthesis of cytotoxic calanone derivatives. Helv. Chim. Acta.

[17] Cao S.G., Sim K.Y., Goh S.H. (1997). Three new coumarins from *Calophyllum teysmannii* var. *inophylloide* (Guttiferae). Heterocycles.

