# Supplementary material for: Identification of Minor Benzoylated 4-Phenylcoumarins from a Mammea neurophylla Bark Extract
Source: Molecules. 2015 Sep 25;20(10):17735–46. doi: 10.3390/molecules201017735 (PMC6332034; doi:10.3390/molecules201017735)
Supplement: Supplementary file 1 [file molecules-20-17735-s001.pdf]

Supplementary Materials

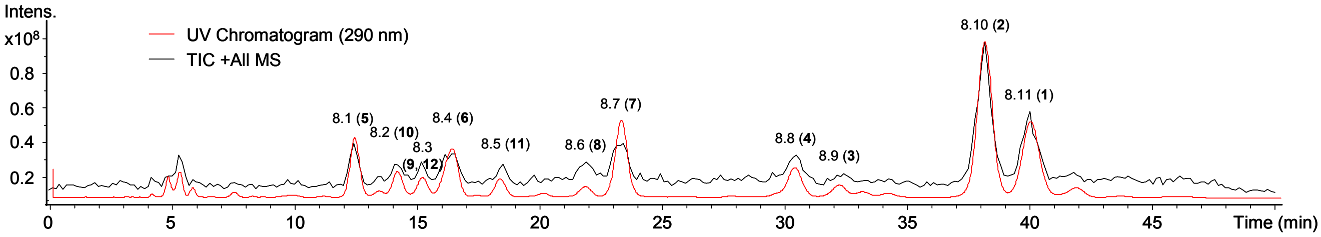

Figure S1. HPLC-UV (290 nm) (red) and total ion current (TIC) from HPLC-ESI$^+$-MS (black) chromatograms for fraction 8 of *M. neurophylla* DCM bark extract.

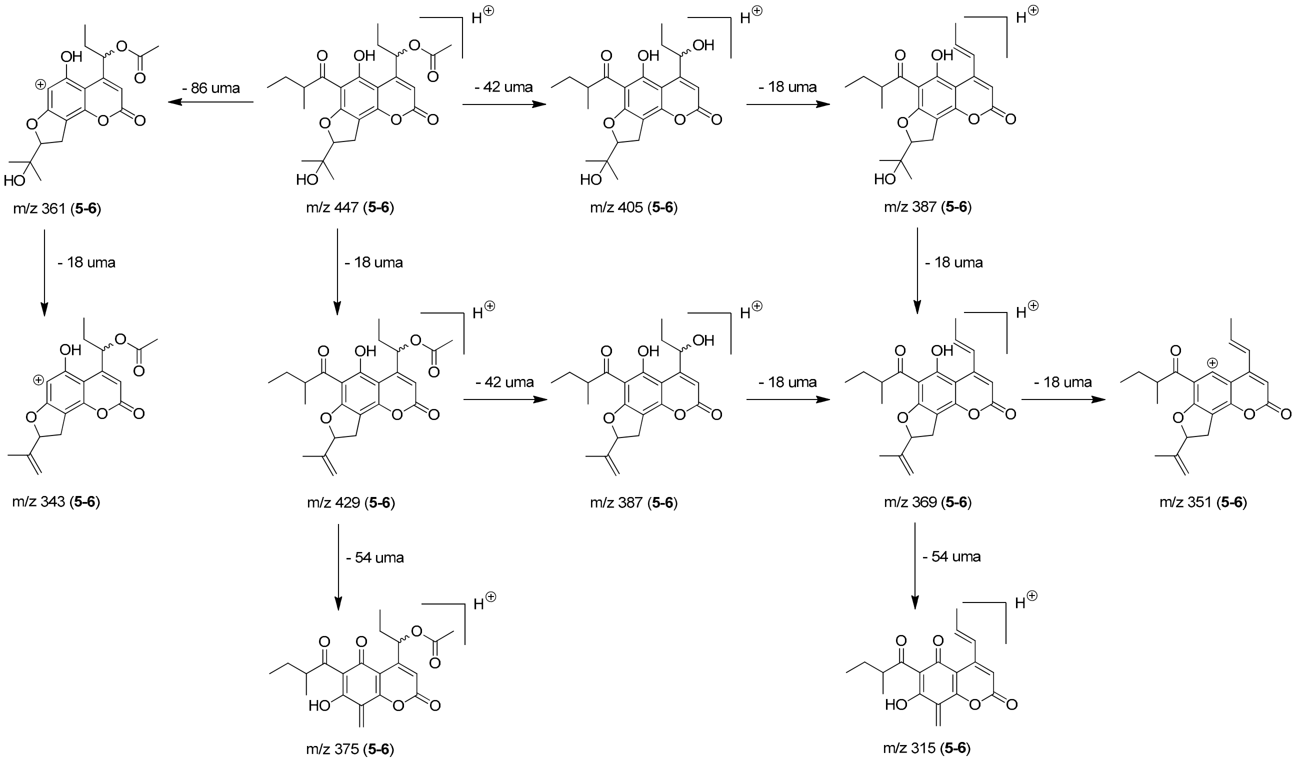

Figure S2. Hypothetical fragmentation pathways for mammea cycloF coumarins **5**–**6**.

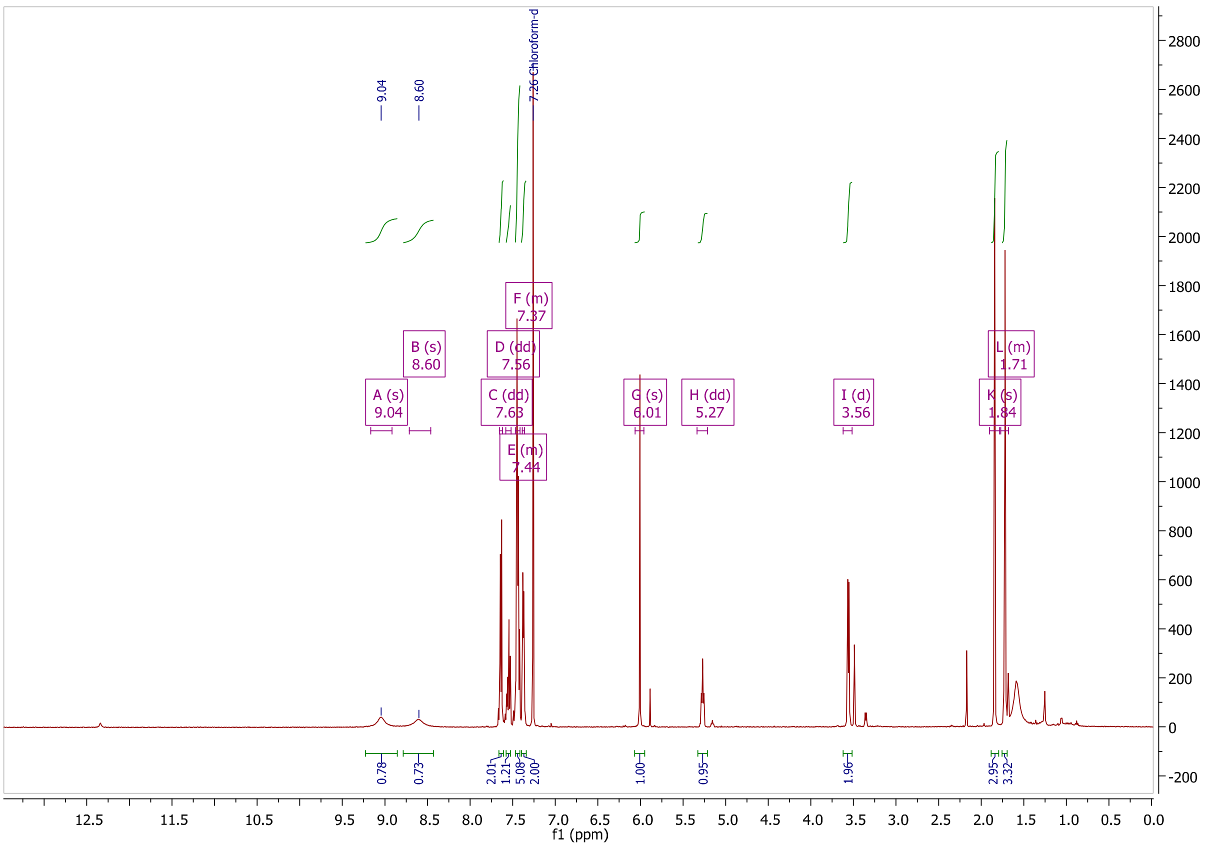

Figure S3. ^{1}H-NMR (500 MHz, $CDCl_3$) spectrum of pedilanthocoumarin B (**7**).

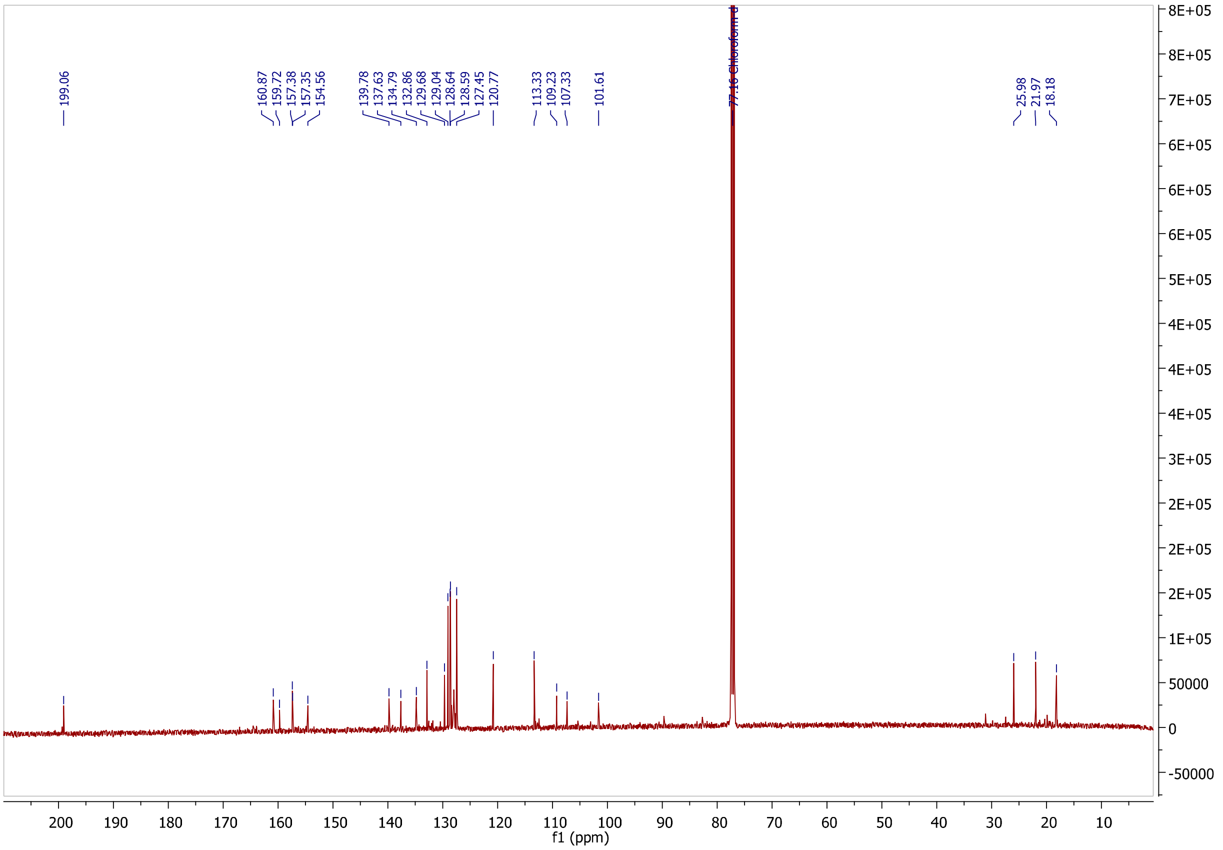

Figure S4. ^{13}C-NMR (125 MHz, $CDCl_3$) spectrum of pedilanthocoumarin B (**7**).

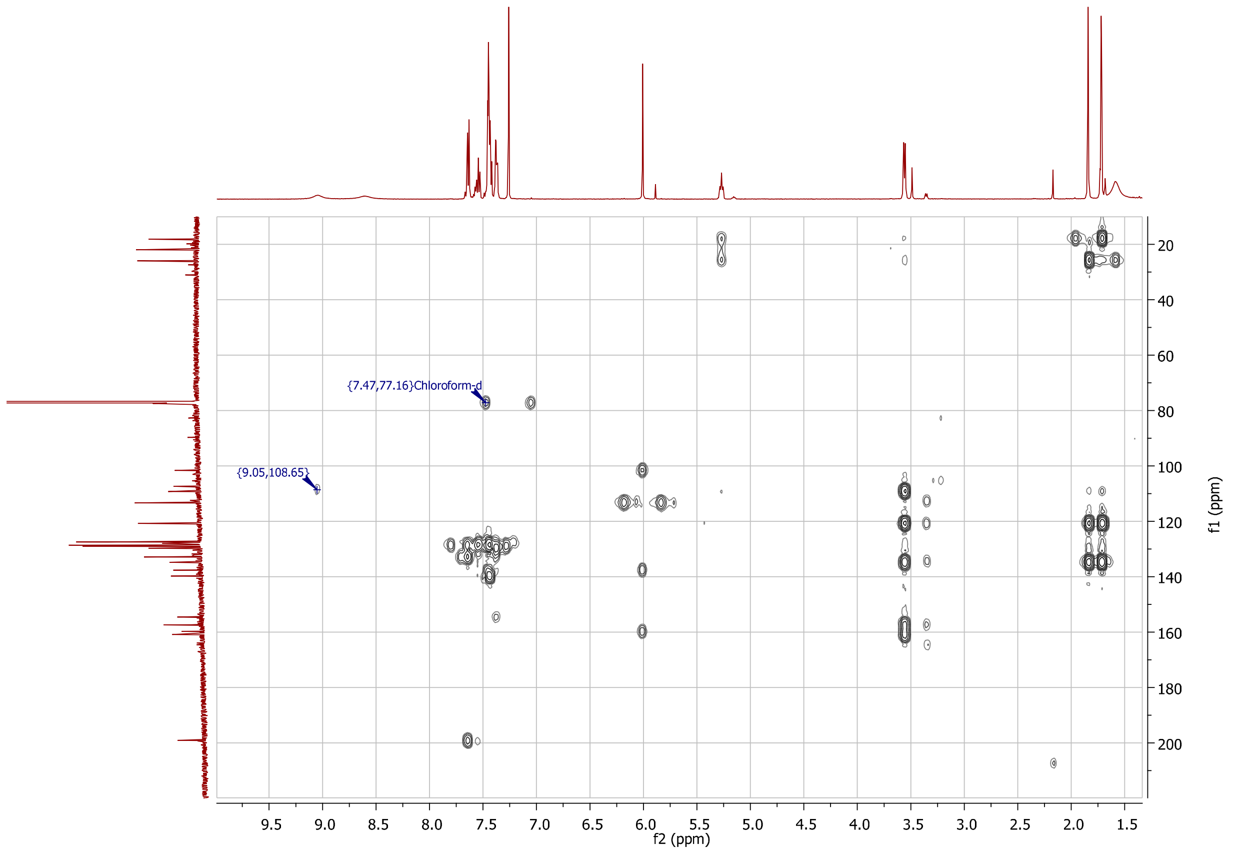

Figure S5. HMBC spectrum (500 MHz, $CDCl_3$) of pedilanthocoumarin B (**7**).

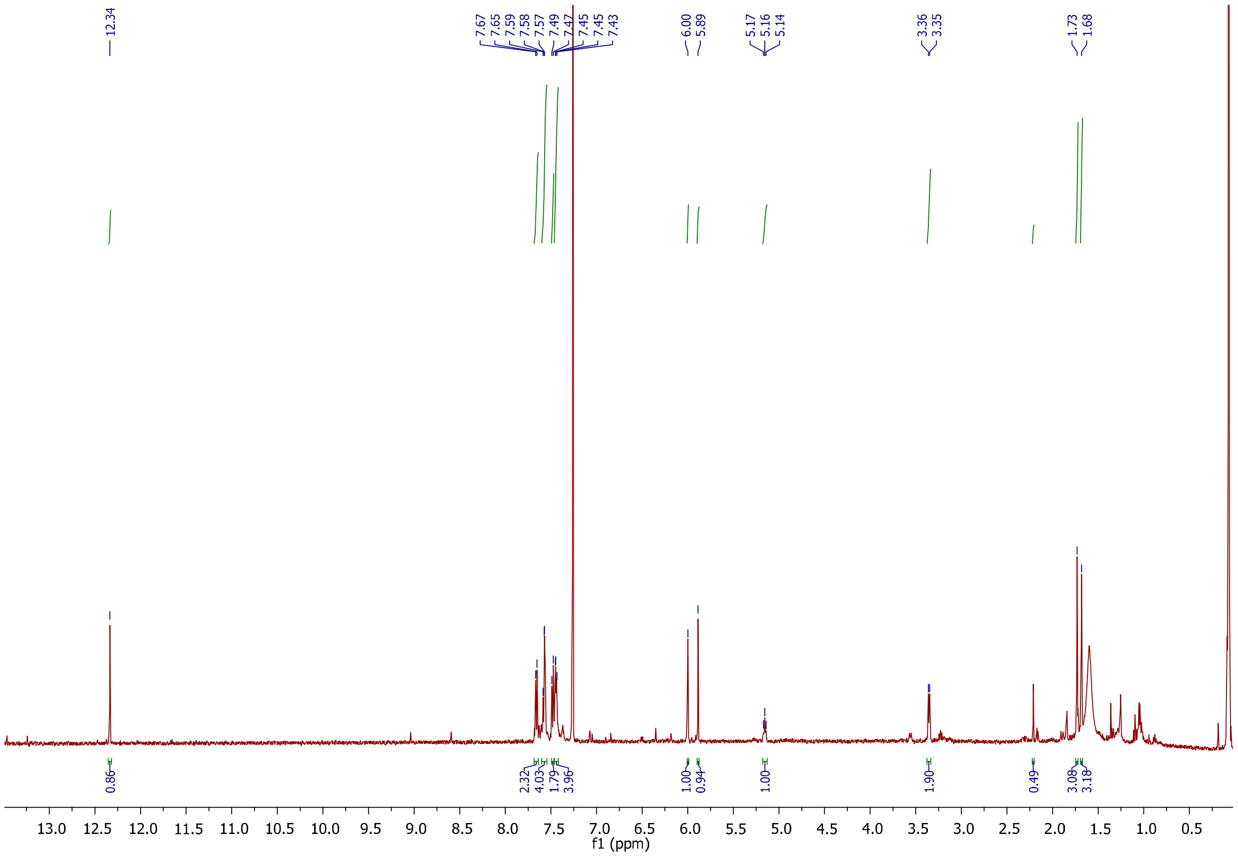

Figure S6. ^{1}H-NMR (500 MHz, $CDCl_3$) spectrum of isopedilanthocoumarin B (**8**).

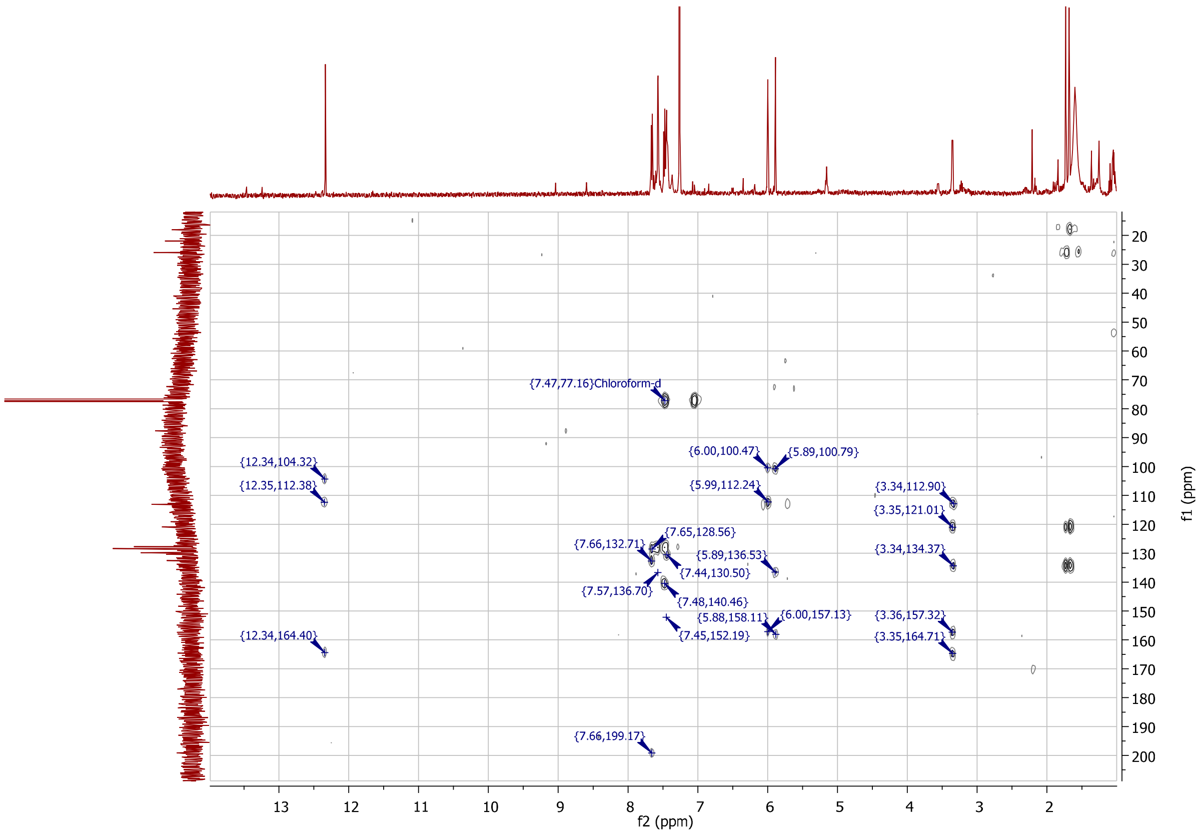

Figure S7. HMBC (500 MHz, $CDCl_3$) spectrum of isopedilanthocoumarin B (**8**).

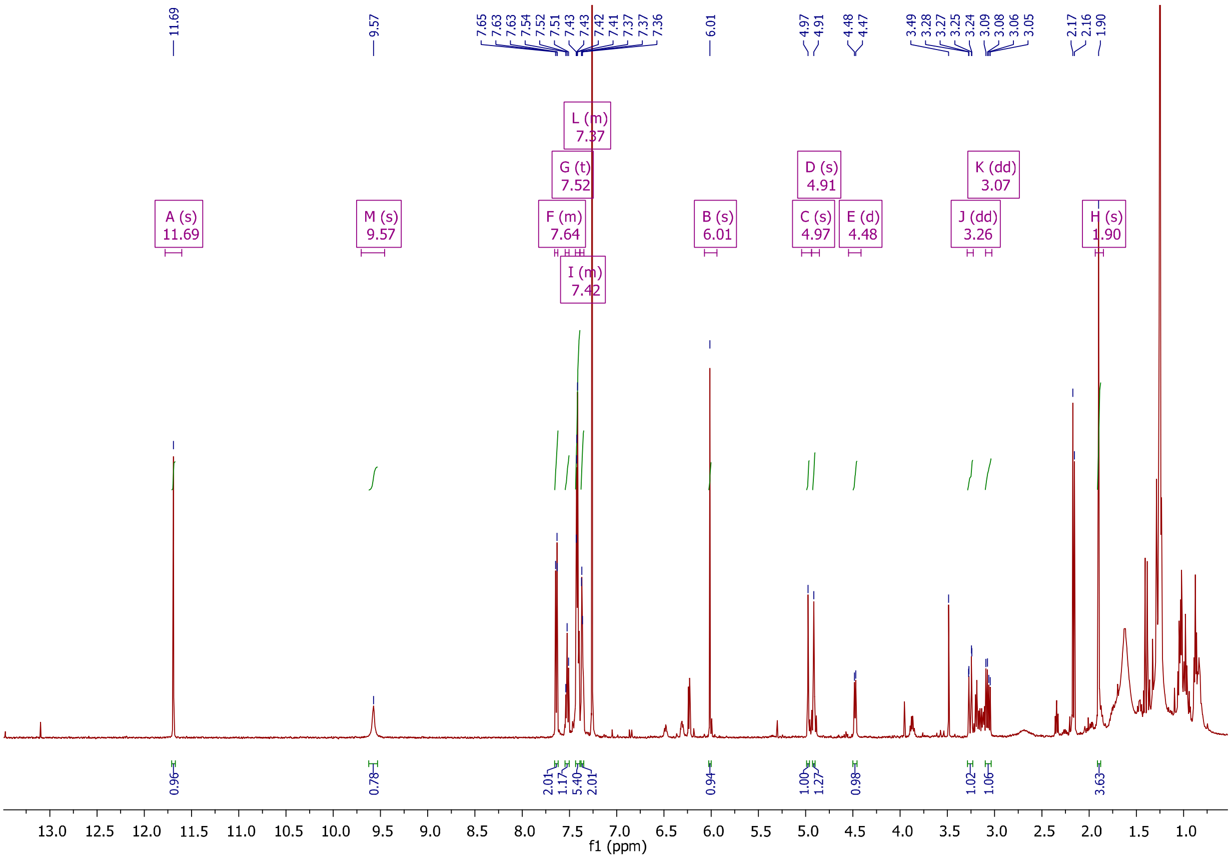

Figure S8. ^{1}H-NMR (500 MHz, $CDCl_3$) spectrum of neurophyllol C (**9**).

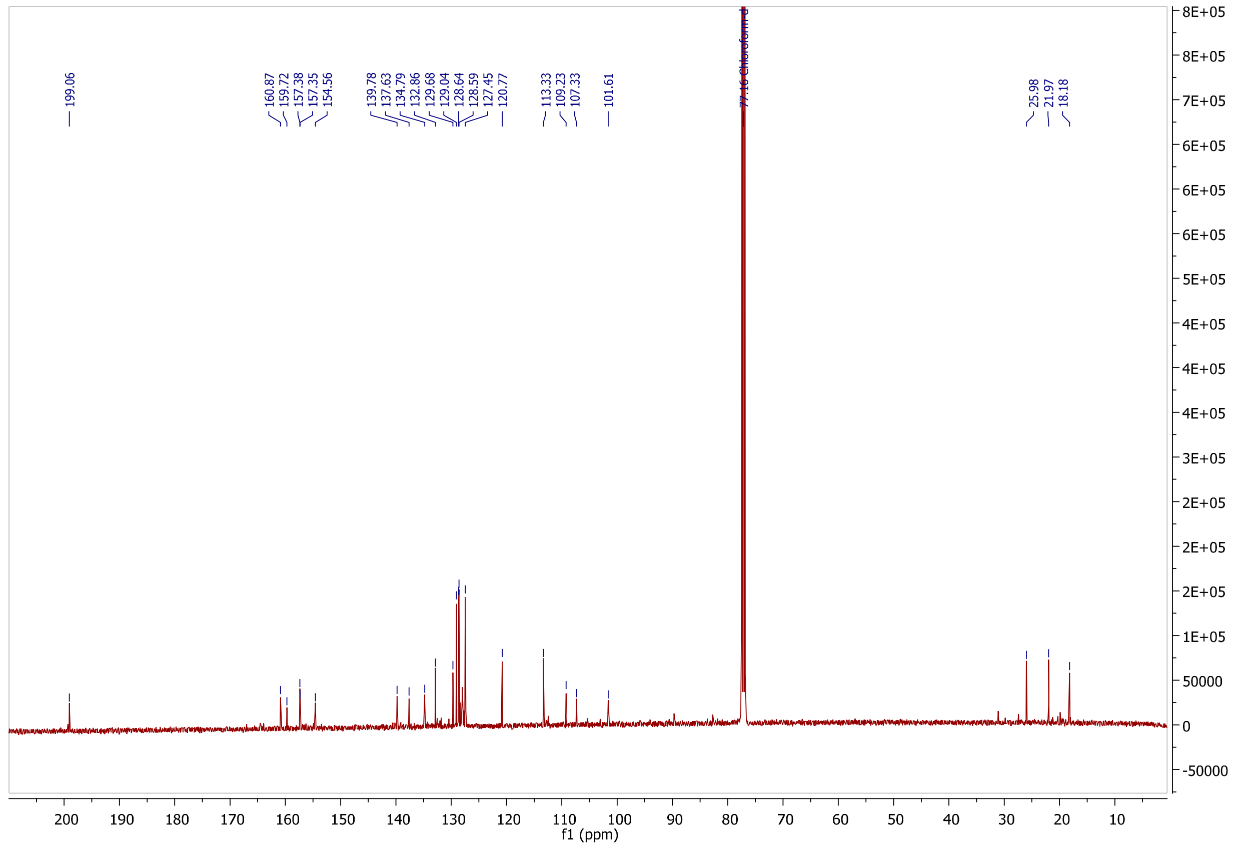

Figure S9. ^{13}C-NMR (125 MHz, $CDCl_3$) spectrum of neurophyllol C (**9**).

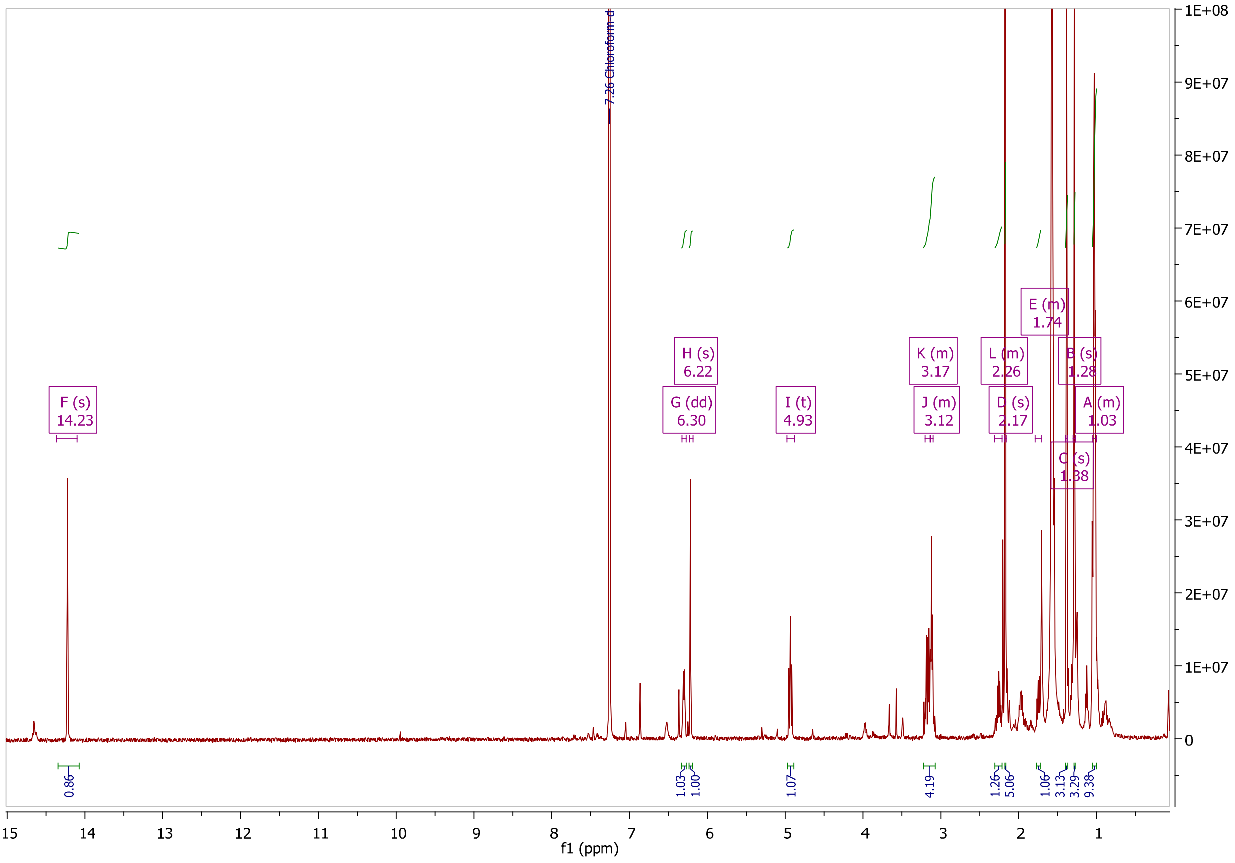

Figure S10. ^{1}H-NMR (500 MHz, $CDCl_3$) spectrum of ochrocarpin H (**10**).

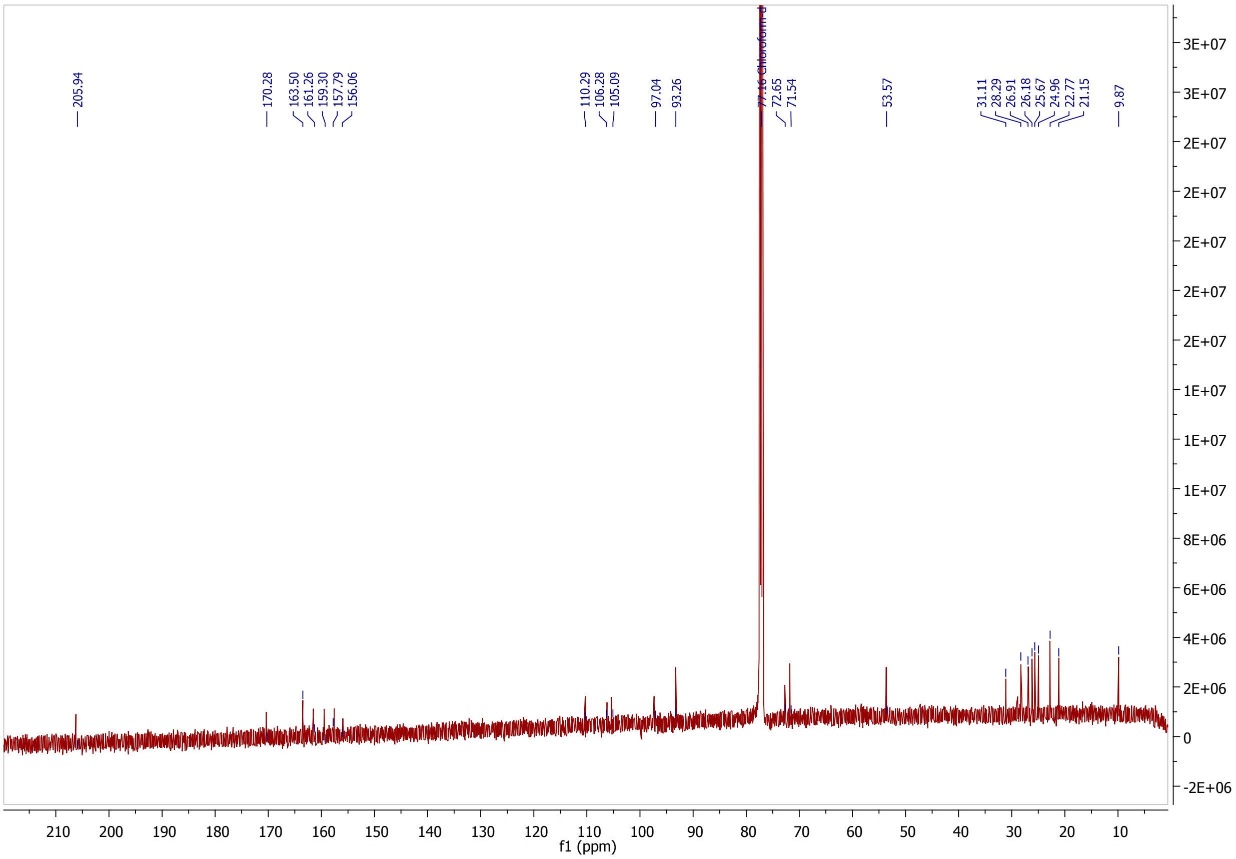

Figure S11. ^{13}C-NMR (125 MHz, $CDCl_3$) spectrum of ochrocarpin H (**10**).

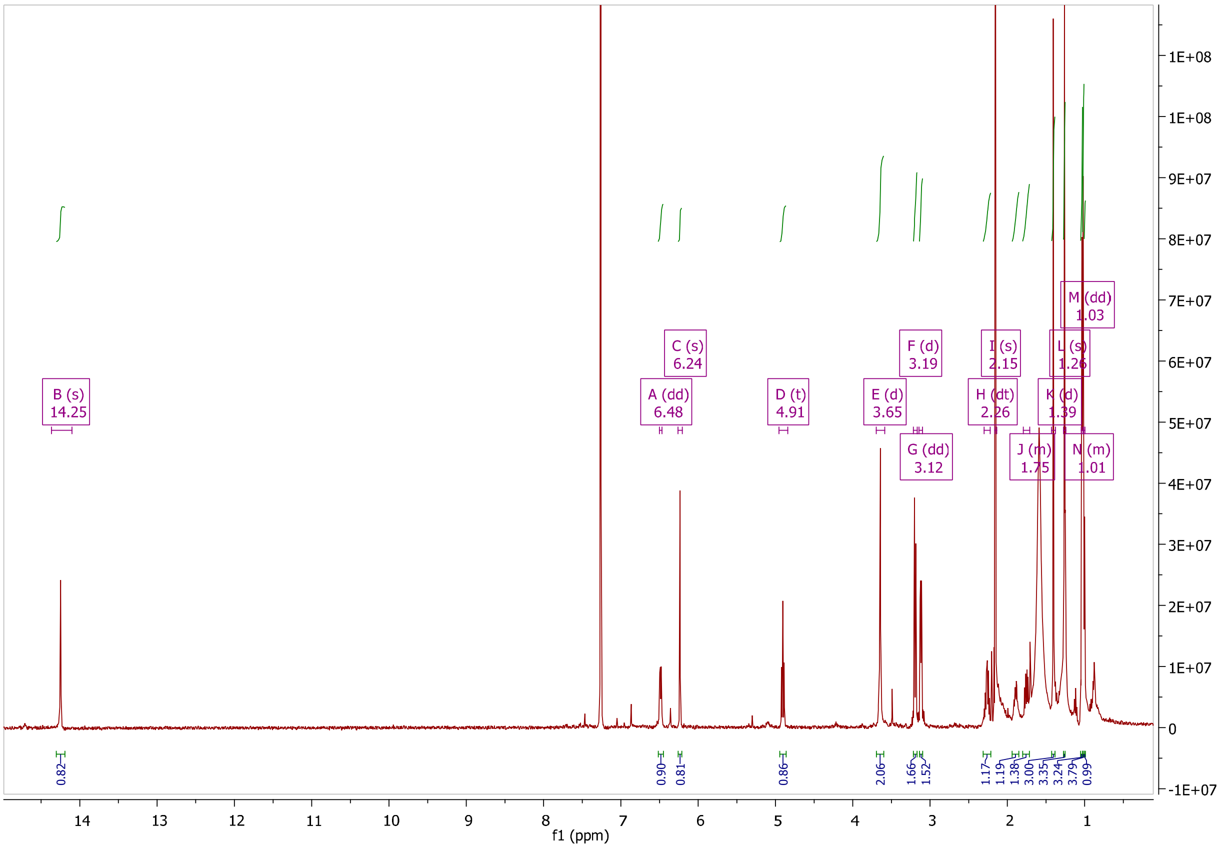

Figure S12. ^{1}H-NMR (500 MHz, $CDCl_3$) spectrum of ochrocarpin I (**11**).

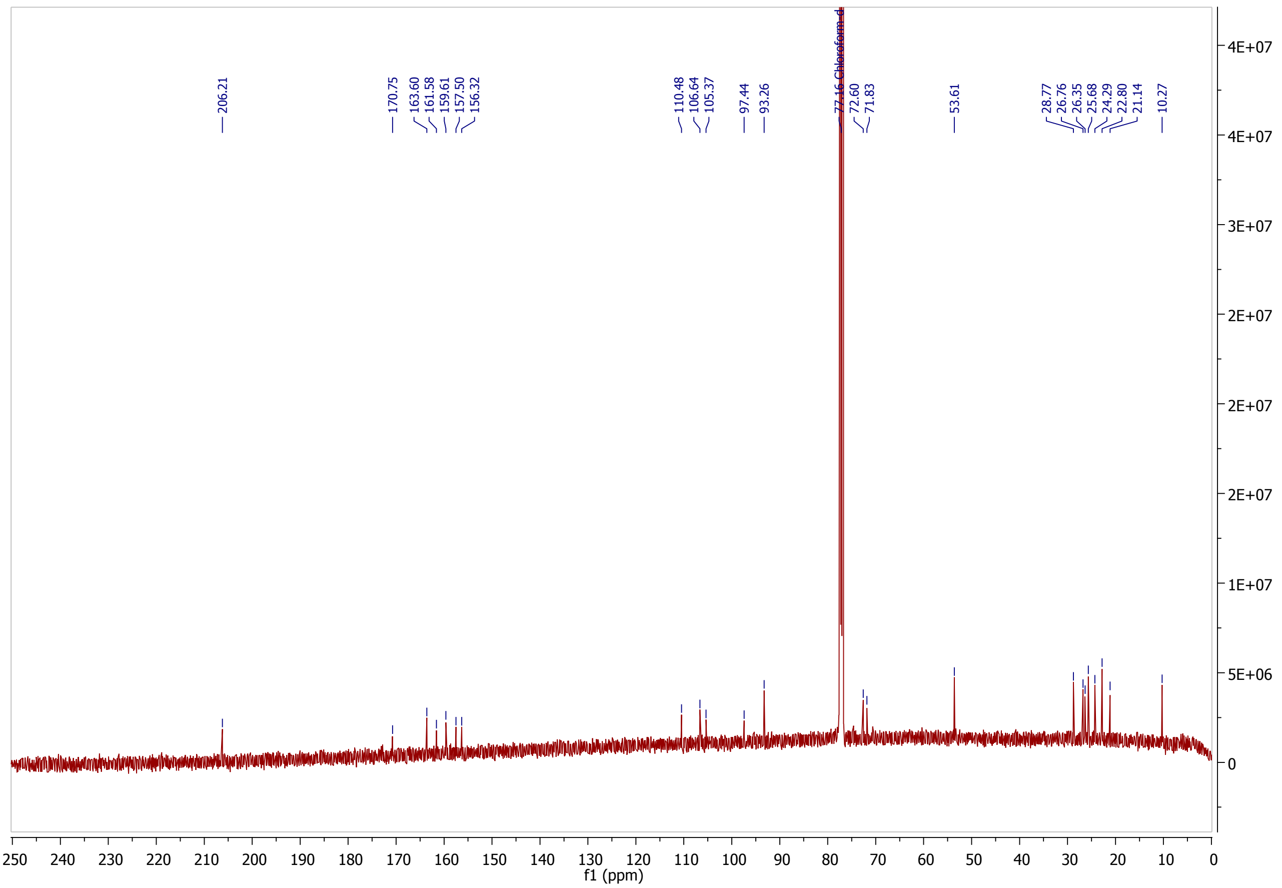

Figure S13. ^{13}C-NMR (125 MHz, $CDCl_3$) spectrum of ochrocarpin I (**11**).
